# Obesity and Risk of Pre- and Postmenopausal Breast Cancer in Africa: A Systematic Review

**DOI:** 10.3390/curroncol32030167

**Published:** 2025-03-14

**Authors:** Najia Mane, Aya Fouqani, Siham Mrah, Majid Omari, Oumnia Bouaddi, Elodie Faure, El Mostafa El Fahime, Sihame Lkhoyaali, Saber Boutayeb, Karima El Rhazi, Chakib Nejjari, Inge Huybrechts, Mohamed Khalis

**Affiliations:** 1 Laboratory of Epidemiology and Research in Health Sciences, Faculty of Medicine, Pharmacy and Dental Medicine, Sidi Mohamed Ben Abdallah University, Fez 30070, Morocco; majid.omari@usmba.ac.ma (M.O.); karima.elrhazi@usmba.ac.ma (K.E.R.); c.nejjari@ueuromed.org (C.N.); 2 Department of Public Health and Clinical Research, Mohammed VI Center for Research and Innovation, Rabat 10112, Morocco; obouaddi@um6ss.ma (O.B.); melfahime@cm6.ma (E.M.E.F.); s.lkhoyaali@gmail.com (S.L.); sboutayeb@cm6.ma (S.B.); 3 Faculty of Medicine and Pharmacy of Rabat, Mohamed V University in Rabat, Rabat 10000, Morocco; aya_fouqani@um5.ac.ma; 4 Laboratory Research of Cancer and Chronic Diseases, Faculty of Medicine and Pharmacy of Tangier, Abdelmalek Essaadi University, Tetouan 93000, Morocco; mrah.siham@etu.uae.ac.ma; 5 Mohammed VI International School of Public Health, Mohammed VI University of Sciences and Health, Casablanca 82403, Morocco; 6 International Agency for Research on Cancer, World Health Organization, 69366 Lyon, France; fauree@iarc.who.int (E.F.); huybrechtsi@iarc.who.int (I.H.); 7 Center of Epidemiology and Population Health, Inserm, UMR 1018, Paris Saclay University, 94805 Villejuif, France; 8 Department of Medical Oncology, National Institute of Oncology, Rabat 6213, Morocco; 9 Euromed Research Center, Euromed University of Fez, Fez 51, Morocco; 10 French Network for Nutrition and Cancer Research (Nacre Network), 78350 Jouy-en-Josas, France; 11 Higher Institute of Nursing Professions and Health Techniques, Ministry of Health and Social Protection, Rabat 10000, Morocco

**Keywords:** breast cancer, obesity, risk, African women, pre-and postmenopausal breast cancer

## Abstract

**Background and Aims**: Several epidemiological studies have investigated the relationship between anthropometric factors and breast cancer (BC), but the results, particularly for premenopausal BC, remain inconsistent and contradictory. The aim of this systematic review is to present an overview of studies examining the association between obesity and BC risk in African women, by menopausal status. **Methods**: PubMed, Scopus, Web of Science, and Google Scholar were searched until 17 February 2025 to identify published articles. The review included original studies, with no restrictions on publication date or language. The exposures studied were height, weight, body mass index (BMI), waist circumference (WC), hip circumference (HC), and waist-to-hip ratio (WHR). The quality of the studies was assessed using the National Institute of Health (NIH). Study selection and data extraction were carried out by two authors separately. **Results**: A total of fifteen case–control studies were included in this systematic review, comprising 45,056 subjects (7221 cases and 37,835 controls). Among them, fourteen studies reported stratified results for pre- and postmenopausal women, and one reported findings for only premenopausal BC. We found that BMI was associated with an increased risk of BC in both premenopausal and postmenopausal women, though the associations varied across studies. Height was associated with an increased risk of pre- and postmenopausal BC. WHR was positively associated with BC in pre- and postmenopausal women, while WC showed a positive association with the risk of postmenopausal BC, and inconsistent results with premenopausal BC. Finally, a higher HC was positively associated with premenopausal and postmenopausal BC. **Conclusions**: The risk of developing BC is higher in obese postmenopausal women. The protective role of BMI has not been demonstrated in African premenopausal women. WHR is a risk factor for premenopausal and postmenopausal BC. There is a need to study the influence of stages of overweight and obesity on BC risk in a large sample of African women in-depth.

## 1. Introduction

Breast cancer is the most prevalent cancer and one of the leading causes of death among women globally. In 2022, it was ranked second in terms of cancer incidence, with almost 2.3 million new cases diagnosed [1]. In terms of mortality, BC is the fourth leading cause of cancer death worldwide, with 666,000 deaths (6.9% of all cancer deaths) [1,2]. Developed countries in North America, Europe, and Oceania have the highest BC incidence rates, while transitioning countries in South America, Asia, and Africa are experiencing a significant increase in incidence and mortality [3,4].

The main factors involved in this multifactorial disease are reproductive factors, lifestyle, and anthropometric characteristics [5,6,7,8]. In view of this burden of disease, the identification of potentially modifiable factors linked to the development of BC takes on a particularly important public health dimension, with obesity/overweight as one of its primary risk factors [9].

According to the latest reports from the World Health Organization (WHO), in 2022, 2.5 billion adults (aged 18 and older) were overweight, with 890 million of them living with obesity [10]. These numbers are expected to increase significantly in the coming decades [11]. Africa is also dealing with the rising challenge of obesity and overweight. According to WHO analysis, adult obesity rates in the ten most affected African countries are projected to range from 13.6% to 31%, while obesity prevalence among children and adolescents is expected to fall between 5% and 16.5% [12].

Anthropometric measurements are frequently used to evaluate overweight and obesity. Recently, there has been a growing prevalence of methods to analyze body shape and size across different age groups [13]. Obesity is primarily assessed using BMI, which is the most commonly used measure, and is generally linked to overall obesity. In contrast, WC and WHR are utilized to evaluate central or intra-abdominal obesity [9]. In addition, HC, which is determined by wrapping a tape measure around the widest part of the buttocks, indicates the amount of adipose tissue present in this area of the body [14].

Several studies and meta-analyses have explored the relationship between anthropometric indices and BC in both premenopausal and postmenopausal women. Overall, there is a consistent positive correlation between BMI and the risk of BC in postmenopausal women [6,15,16,17,18,19]. A recent pooled analysis of 13 cohort studies revealed that higher BMI was associated with an increased risk of BC in postmenopausal women [20].

On the other hand, conflicting findings persist regarding premenopausal BC, particularly BMI and other anthropometric measures [21,22]. The World Cancer Research Fund (WCRF) and the American Institute for Cancer Research (AICR) report presented strong evidence indicating that being overweight or obese was associated with a reduced risk of premenopausal BC [23]. In addition, more recent studies have revealed these findings [24,25]. A meta-analysis involving 7930 premenopausal patients found that for every 5 kg/m^2^ increase in BMI, the risk of BC was reduced by approximately 8% [6]. Some studies have indicated a negative correlation or non-significant inverse association [26,27], while others have reported no relationship [17,18]. In contrast, other studies and meta-analyses have shown a modest to strong positive association between obesity and the risk of premenopausal BC [20,28,29].

Recent studies have found that WC and WHR are associated with an increased risk of BC in postmenopausal women [30,31], a reduced risk in premenopausal women [21], or no significant association [32]. A meta-analysis accounting for adjusted BMI revealed an association between WC and the risk of premenopausal BC, while WHR showed no association with premenopausal BC or with postmenopausal BC [9]. Recently, a large meta-analysis including 26 case–control studies and 31 prospective studies demonstrated a positive association between WC, WHR, and BC; subgroup analysis also revealed that central obesity (WC and WHR) is associated with a higher risk of BC in both premenopausal and postmenopausal women [33].

Despite numerous research findings, the link between obesity and BC risk remains controversial, especially in obese premenopausal women, where the results are still unclear and inconsistent [20]. Notably, several African countries have reported the highest BC mortality rates globally [34], whereas the findings of systematic reviews showed that obesity was more prevalent in urban areas than in rural ones, with a significant rise in obesity rates among African women. Furthermore, levels of inflammatory markers associated with various co-morbidities like BC were significantly higher in Africans than in Caucasians [35]. These findings have prompted us to conduct this systematic review.

To our knowledge, no systematic review has been carried out to examine the relationship between obesity and BC risk in native African women. Therefore, the present systematic review aims to present an overview of studies investigating associations between obesity and BC risk in Africa, while elucidating this relation.

## 2. Materials and Methods

This systematic review is reported according to the 2020 PRISMA (Preferred Reporting Items for Systematic Review and Meta-Analysis) guidelines [36]. The PRISMA checklist is provided in the Supplementary Materials (Table S1).

### 2.1. Protocol and Registration

The review protocol was registered on 25 March 2024 in the International Prospective Register of Systematic Reviews, “PROSPERO” (registration number CRD527649).

### 2.2. Data Sources and Search Strategy

We developed a detailed search strategy, and conducted an extensive search of various databases to locate relevant studies: PubMed, Scopus, Web of Science, and Google Scholar. No limitations of date and no language restrictions were applied if translations were available. For a more exhaustive search, we reviewed the references cited in the identified articles, as well as those from the prior review and meta-analysis investigating the association between anthropometric factors and breast cancer, to identify other articles not found in the databases consulted.

The search strategy used was based on Boolean operators according to the database to be indexed, and Medical Subject Headings (MeSH) terms for each variable. We also created alerts in these databases corresponding to our research equation in order to receive articles that met the inclusion and exclusion criteria for our review.

We searched for (breast cancer OR breast neoplasms OR breast tumor OR breast adenocarcinoma OR breast carcinoma OR mammary cancer OR cancer of breast OR breast malignant neoplasms OR premenopausal breast cancer OR postmenopausal breast cancer) AND (Obesity OR adiposity OR body weight OR fat OR obese OR body mass index OR BMI OR body mass OR body size OR overweight OR over-weight OR waist hip ratio OR body fatness OR body fat) AND (Africa OR Africa south of the Sahara OR South Africa OR Africa western OR Africa southern OR Africa northern OR Africa eastern OR Africa central OR North Africa OR Sub-saharan africa), and the name of each African country.

Our search began on 16 November 2023 and updated on 17 February 2025, in PubMed (all fields, 1711 results, with the filter “humans”), Scopus (title/abstracts/keywords, without any filters, 979 results), Web of Science (all fields, without any filter, 990 results) and Google Scholar (1300 results, filtered by title and abstract) (Table S2 in Supplementary Materials).

### 2.3. Inclusion and Exclusion Criteria

This study’s eligibility criteria, following the PICOS (Population, Intervention, Comparison, Outcome and Study Design) methodology, included studies containing information on BMI, height, weight, and other anthropometric indicators of adiposity: HC, WC, and WHR, in relation to BC risk (premenopausal and postmenopausal), providing association measurements and sufficient data on anthropometric measurements.

Our exclusion criteria were studies of male BC; cross-sectional studies; studies without original data such as literature reviews; books; editorials; case reports; abstracts; letters; conference abstracts and commentaries; studies for which the full text is not available and which have not been peer-reviewed; studies that combine pre- and postmenopausal BC (without presenting stratified results); studies on the prognosis, treatment, mortality, and survival outcomes of BC, particularly with regard to BC recurrence related to overweight or obesity, and associations between BC and obesity or overweight in childhood or adolescence, or birth weight.

### 2.4. Study Selection and Data Collection Process

References were exported into the reference manager tool “Zotero” to manage duplicate entries. Two reviewers (N.M. and A.F.) independently screened all titles and abstracts identified by the search for relevance to the review question, according to eligibility criteria by using the PRISMA diagram flow for selection. Disagreements between reviewers were resolved by a third reviewer (M.K.).

### 2.5. Data Extraction and Items

Regarding the included studies, two authors (N.M. and A.F.) independently extracted information about characteristics of the studies and their results, which was checked by M.K., including title; first author; year of publication and country; period of enrollment; study type; number of cases; number of controls; outcome investigated; method of data collection; statistical method; anthropometric measurement; type of anthropometric measurement (self-reported or directed measured); results; variables of adjustment; and limitations (Table 1).

### 2.6. Quality Assessment

Three authors (N.M., A.F. and M.O.) assessed the quality of the included studies independently. The quality of the studies was assessed using the National Institute of Health (NIH) Quality Assessment Tool, which is a standardized tool designed to evaluate the internal validity of various study designs. This tool assesses key aspects such as study objectives, population selection, sample size justification, outcome measurements, and potential confounding variables [37]. For each study, various variables influencing the quality of observational studies were evaluated. Elements assessed included the presentation of key study design components, eligibility criteria, exposures clearly defined (obesity and overweight), outcomes clearly specified (pre- and postmenopausal BC), the representativeness of the cases and controls in the representative population, details of the exposure measurement, statistical methods, and adjustment for potential confounding variables (Table S3: NIH Quality Assessment Tool for case-control Studies in the Supplementary Materials).

**Table 1:** Main characteristics of the included studies.

Author, Year and Country	EnrollmentPeriod	Study Type	Sampling Size of Case/ ControlParticipants	Materials/Method of Data Collection	Outcomes	Statistical Methods	Results	Adjustment Variables	Limitations
Laamiri FZ et al., 2016, Morocco [38]	2008–2010	Case–control	124/148	-Basic questionnaire -Method of measuring outcome was not mentioned	BMI	Univariate logistic regression	-BMI: preM BC (OR = 0.994; 95%CI = 0.937–1.05; *p* = 0.849).	Age	
Adebamowo CA et al., 2003, Nigeria [39]	1998–2000	Case–control	234/273	-Face-to-face interviews by trained nurse -Method of measuring outcomes was not mentioned	BMI, height, weight, WC, WHR, HC	Multivariable logistic regression	-WHR: postM BC (aOR = 2.67; 95% CI = 1.05–6.80; *p* = 0.04), and preM BC (aOR = 1.80; 95%CI = 0.85–3.81; *p* = 0.13).	Age, height	Selection and recall bias, absence of information about breastfeeding and the features of a “Western lifestyle”
Adebamowo CA et al., 2003, Nigeria [40]	1998–2000	Case–control	234/273	-Questionnaire -Outcomes were measured directly by trained nurse	BMI, height, weight	Multivariable logistic regression	-BMI ≥ 30 kg/m^2^: preM (aOR = 1.21; 95%CI = 0.56–2.60; *p* > 0.05), and postM BC (aOR = 1.82; 95%CI = 0.78–4.31; *p* > 0.05). -Increasing height: preM (aOR = 1.05; 95%CI = 1.01–1.10; *p* = 0.05), and postM BC (aOR = 1.07; 95%CI = 1.01–1.13, *p* = 0.05).	Age, age at onset of menarche, regularity of periods, social status, later age at first full-term pregnancy	
Okobia MN et al., 2006, Nigeria [41]	2002–2004	Case–control	250/250	Unknown	WHR, BMI, height	Conditional logistic regression	-WHR:preM (aOR = 2.56; 95%CI = 1.48–4.41; *p* < 0.05), and postM BC (aOR = 2.00; 95%CI = 1.04–2.53; *p* < 0.05). -Increasing height: preM BC (aOR = 1.59; 95% CI = 0.98–2.58), and postM BC (aOR = 1.08; 95%CI = 0.62–1.89). -BMI and weight: not associated with risk of BC in preM and postM BC; WC and HC were not significant predictors of BC risk in preM women.	Age	Recall bias, use of hospital controls, and recruitment of both prevalent and incident cases
Ogundiran TO et al., 2010, Nigeria [42]	1998–2009	Case–control	1233/1101	-Structured questionnaire administered -Outcomes were measured directly by research nurses	Height, weight, BMI	Logistic regression	-Height: preM BC (OR = 2.11; 95%CI = 1.46–3.05; *p* < 0.001), postM BC (OR = 1.75; 95%CI = 1.06–2.88; *p* < 0.002). -BMI ≥ 28 kg/m^2^: preM BC (OR = 0.70; 95%CI = 0.50–0.98; *p* = 0.027), and postM BC (OR = 0.76; 95% CI = 0.48–1.21; *p* = 0.15). -Weight: preM (OR = 0.78; 95% CI = 0.55–1.12; *p* = 0.27) and postMBC (OR = 0.90; 95% CI = 0.57–1.44; *p* = 0.48).	Age at diagnosis, age at menarche, menopause, ethnicity, education, number of live births, age at first live birth, duration of breastfeeding, menopausal status, family history of BC, benign breast disease, hormonal contraceptives, alcohol and height	Cases were older than controls, weight was not recorded in early life such as at the age 18 years, the majority of subjects are preM BC women, limited power to assess the relation of weight and postM BC risk
Ogundiran TO et al.,2012, Nigeria [43]	1998–2009	Case–control	1233/1101	-Structured questionnaire -Outcomes were measured directly by research nurses	BMI, height, weight, WHR, WC, HC	Logistic regression models	-WC: preM BC (aOR = 2.40; 95%CI = 1.52–3.78; *p* < 0.001), and postM BC (OR = 2.21; 95%CI = 1.25–3.91; *p* < 0.001). -WHR: preM BC (aOR = 2.12; 95% CI = 1.49–2.99; *p* < 0.001), postM BC (aOR = 2.26; 95% CI = 1.39–3.68; *p* < 0.001). -HC: inverse association with preM BC (aOR = 0.35; 95%CI = 0.22–0.56; *p* < 0.001), and postM BC (aOR = 0.38; 95%CI = 0.22–0.66; *p* < 0.001).	Age at diagnosis or interview, ethnicity, education, age at menarche, number of live births, age at first live birth, duration of breastfeeding, first-degree family history of BC, benign breast disease, hormonal contraceptives, alcohol, menopausal status, height, HC, BMI, WC	Cases were significantly older than controls, residual biases and confounding from variables that we did not collect, inaccurate recall, WC, HC and WHR are indirect measures of abdominal visceral fat
JordanI et al., 2013, Tanzania [44]	2004–2007	Case–control	115/230	-Interview by standardized and pre-tested questionnaire -Outcomes were measured directly by trained nurse	BMI at 20 years, BMI at interview	Logistic regression models	-Higher BMI at age 20 years:preM BC (aOR = 1.41; 95%CI = 1.10–1.81; *p* = 0.01), and postM BC (aOR = 1.38; 95%CI = 1.06–1.80; *p* = 0.02). -Higher BMI at interview: no association with preM and postM BC.	Age, place of living	
Wang S et al., 2018, Nigeria [45]	1998–2015	Case–control	1811/2225	-Structured questionnaire administered -Outcomes were measured directly by research nurses	BMI	Multivariable logistic regression	-BMI ≥ 30 kg/m^2^: preMBC (aOR = 0.71; 95%CI = 0.57–0.89; *p* = 0.001), postM BC (aOR = 0.68; 95%CI = 0.52–0.89; *p* < 0.001).	Age	Model developed and validated in same population, model may not perform well in other African populations, lack of information on other predictors, incompleteness in case reporting in the Ibadan Cancer Registry, same incidence and mortality
Khalis M et al., 2020, Morocco [46]	2016–2017	Case–control	300/300	-Face-to-face depth questionnaire -Outcomes were measured directly and through self-reporting	BMI, height, weight, WHR, WC, HC, young-adult BMI, weight gain since the age of 20, body silhouettes, trajectories	Unconditional logistic regression	-Higher WC and HC: preM (aOR = 2.92; 95%CI = 1.33–6.42; *p* < 0.01), (aOR = 3.00; 95%CI:1.42–6.33; *p* = 0.01), and post M BC (aOR = 4.46; 95%CI = 1.86–10.66; *p* < 0.01), (aOR = 4.08; 95%CI:1.76–9.42; *p* < 0.01). -Body shape at younger ages (6–11 years) was inversely associated with preM BC (aOR = 0.31; 95%CI = 0.12–0.80; *p* = 0.01), and postM BC; (aOR = 0.40; 95%CI = 0.15–1.07; *p* = 0.05). -Greatest increase in body shape trajectory had higher risk for both preM (aOR = 2.74; 95%CI = 1.03–7.26; *p* < 0.01), and postM BC (aOR = 3.56; 95%CI = 1.34–9.44; *p* < 0.01). -BMI > 30 kg/m^2^, height, weight, WHR, young-adult BMI (kg/m^2^), and weight gain since the age of 20 were not significantly associated with BC risk in either preM or postM BC.	Age, area of residence, wealth score number of live births, history of oral contraceptives, history of breastfeeding, age at first full-term pregnancy, physical activity, current BMI	Small sample size, self-report, current body size of our cases may have been affected by the disease, or its symptoms, prior to BC diagnosis
Brandão M et al., 2021, Mozambique, Sub- SaharanAfrica [47]	2014–2017	Case–control	138/638	-Face-to-face interviews -Outcomes were measured directly	Height, weight, and BMI	Multivariable logistic regression	-Higher weight and BMI: postM BC (per 1 kg increase: aOR = 1.05; 95%CI, 1.02–1.08; *p* ≤ 0.001), (per 1 kg/m^2^ increase: aOR = 1.11; 95%CI = 1.04–1.18; *p* ≤ 0.001), preM BC (aOR = 0.98; 95%CI = 0.96–0.99; *p* < 0.001), (aOR = 0.95; 95%CI = 0.91–0.99; *p* < 0.001). -Height: postM (aOR = 1.87; 95%CI = 1.13–3.10; *p* = 0.101).	Province, age, education, BMI, menopausal status, height, number of live births	Some missing data, smaller sample size, 3/4 cases had advanced BC at the time of diagnosis
Akinyemiju T et al., 2021, Nigeria [48]	2015–2017	Case–control	419/286	-Questionnaire -Outcomes were measured directly by trainedresearchstaff	Height, weight, and BMI	Logistic regression models	In preM/perimenopausal, but not postM women, both higher BMI and weight were significantly associated with reduced risk of BC.	Age, age at menarche, number of pregnancies and births, menopausal status, and prior hypertension diagnosis, BMI, height, weight	Recall bias, BMI, height and weight were recorded at the time of diagnosis: unable to rule out the possibility of reverse causality
Kamal RM et al., 2022, Egypt [49]		Case–control	275/30,168	-Face-to-face interview -Outcomes were measured directly by administration staff and technologists	BMI	Logistic regression model	-BMI (≥25): negative insignificant difference with preM BC (aOR = 0.877; 95%CI = 0.354–2.170; *p* = 0.776), and statistically significant positive difference with postM BC (aOR = 2.280; 95%CI = 1.071–4.862; *p* = 0.028).		
Jacobs I et al., 2022, South Africa [50]	2014–2017	Case–control	396/396	-Face-to-face interviews -Outcomes were measured directly	BMI, weight, height, WC	Multivariate logistic regression	-Smaller WC: postM BC(aOR = 1.69; 95% CI = 1.08–2.63; *p* = 0.020), and preM BC (aOR = 1.30; 95%CI = 0.69–2.44; *p* = 0.406). -BMI: preM (aOR = 1.01; 95%CI = 0.56–1.81; *p* = 0.978), postM BC (aOR = 1.18; 95%CI = 0.76–1.83; *p* = 0.454).	Total energy intake, individual income/month, ethnicity, level of education, physical activity, WC, alcohol, breastfeeding, menopausal status	Limited sample size, no physical examination for control participants, information bias, homogeneity of the study population for some of the individual WCRF/AICR
Mohammed AM et al., 2023, Egypt [51]	2020–2021	Case–control	112/112	-Face-to-face interview -Medical records -Outcomes were Measured directly	BMI, WC, weight	Logistic regression	-BMI: preM BC (aOR = 1.406; 95%CI = 1.194–1.656; *p* < 0.001). -WC: preMBC by 8.6% (aOR = 0.914; 95%CI = 0.868–0.963; *p* = 0.001).		
Oyamienlen CS et al., 2019, Nigeria [52]	2014–2016	Case–control	347/334	-Structured questionnaire -Method of measuring outcome was not mentioned	BMI, height, weight	Logistic regression	BMI ≥ 30 kg/m^2^**:** preM BC (OR = 2.210; 95%CI = 1.246–5.970; *p* = 0.120), and postM BC (OR = 2.720; 95% CI = 1.204–4.054; *p* = 0.010).		Resultscannot be generalized to all women in Nigeria, did not explore stagingof BC

Abbreviations: breast cancer (BC); body mass index (BMI); waist circumference (WC); waist-to-hip ratio (WHR); hip circumference (HC); odds ratio (OR); adjusted odds ratio (aOR); confidence interval (CI); premenopausal breast cancer (preM BC); postmenopausal breast cancer (postM BC); World Cancer Research Fund (WCRF); American Institute for Cancer Research (AICR).

## 3. Results

**Figure 1:**
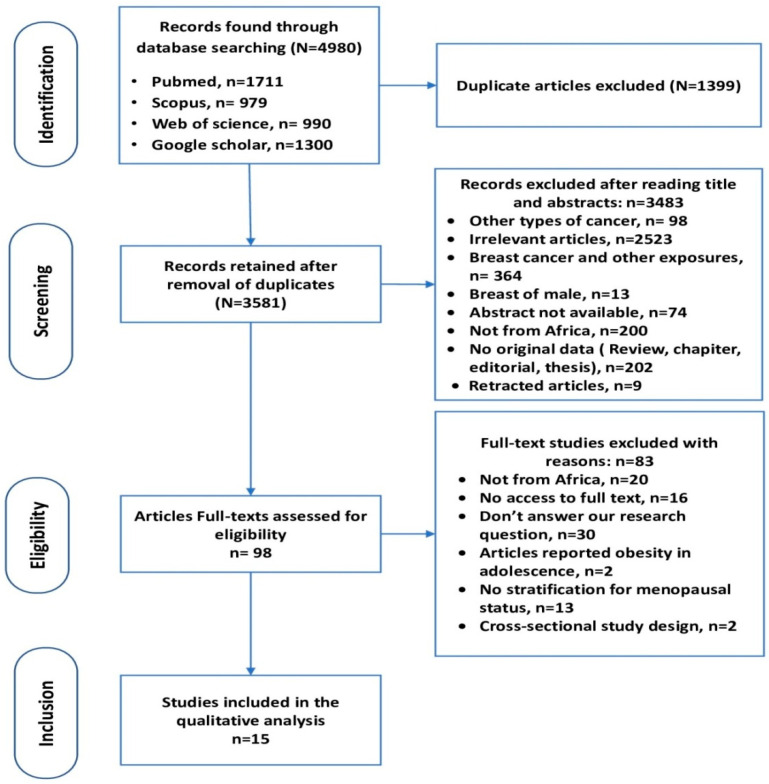
PRISMA flow diagram for the studies selected.

We identified 4980 results. A total of 3581 documents were retained after the elimination of duplicate data, and 3483 articles were excluded after the screening of titles and abstracts. The eligibility of 98 full-text articles was assessed, leading to the exclusion of 83 studies for various reasons. Finally, 15 articles that fully met the inclusion criteria were retained. Figure 1 shows a flow diagram of the process of this systematic review search, in accordance with the PRISMA guidelines.

### 3.1. Study Characteristics

The fifteen studies included are all case–control studies, were published in English, and ranged from 2003 to 2023, with a total of 45,056 participants (7221 cases and 37,835 controls). One study provided information solely on premenopausal BC [38]. Fourteen studies provided stratified results of premenopausal and postmenopausal BC [39,40,41,42,43,44,45,46,47,48,49,50,51,52].

**Figure 2:**
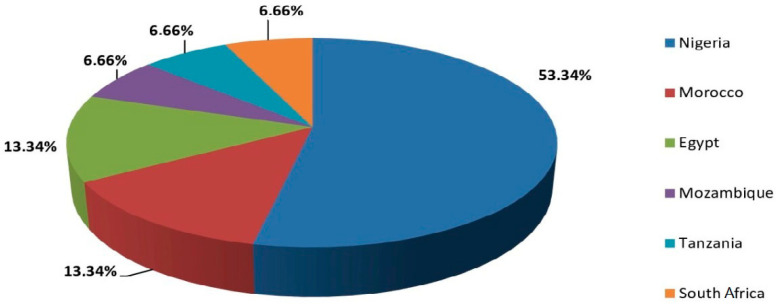
Countries of included studies.

Of the fifteen studies included, eight were conducted in Nigeria [39,40,41,42,43,45,48,52], two in Morocco [38,46], and two in Egypt [49,51]. The remaining studies were more geographically dispersed, with one each from Tanzania, Mozambique, and South Africa [44,47,50], as illustrated in Figure 2.

### 3.2. Reported Outcomes

Among the included studies, three studies evaluated the association between BC and BMI [38,45,49,52], two studies provided data on the relationship between BC and BMI, height, and weight [40,42], and two studies presented results solely on the relationship of BC with weight and BMI) [47,48]. One study provided data concerning the association of BC with current BMI, weight, height, WC, HC, WHR, early-life-reported silhouette, trajectories of body size, young-adult BMI, and weight gain since the age of 20 [46], while two studies provided data on the relationship of BC with BMI and WC [50,51]. Only one study presented results focusing on the relationship between BC and all measurements of central obesity (WC, WHR, and HC) [43], whereas one evaluated the association between WHR and BC [39], and one assessed WHR and height [41]. Finally, one study presented findings on the relationship between BC and BMI at 20 years [44].

The adjustment variables were different in each study, with most articles offering risk estimates after adjustment, with odds ratios adjusted for age at diagnosis, age at interview, age at first menarche, menstrual regularity, social status, later age at first full-term pregnancy, ethnicity, education, age at first live birth, duration of breastfeeding, menopausal status, family history of BC, benign breast disease, first-degree family history of BC, area of residence, wealth score, history of oral contraceptives, age at first full-term pregnancy, physical activity, number of pregnancies, number of births, previous diagnosis of hypertension, total energy intake, alcohol, individual income/month, height, weight, current body mass index, waist circumference, and hip circumference.

The characteristics of each study are summarized in Table 1.

Below, we present the findings of the association between obesity and BC by menopausal status.

### 3.3. Obesity and Breast Cancer Risk in Premenopausal Women

Nine studies reported an elevated risk of BC associated with various measures, including higher height [40,42], higher weight [47], WHR [41,43], WC [43,46], higher BMI at 20 years [44], BMI [51], BMI ≥ 30 kg/m [52], HC, and greatest increase in body shape trajectory [46]. Conversely, four studies indicated a decreased risk of BC in relation to higher weight [47,48], WC [51], and BMI ≥ 30 kg/m [45,47,48]. Additionally, six articles found no significant association between BC and measures like BMI [49,50], BMI ≥ 30 kg/m^2^ [40,42,46,49], height [41,46], weight [41,42,46], WC [41,46,50], and HC [41]. Furthermore, six articles reported no association between BC and BMI [38,41,44], higher BMI at interview [44], WHR [39,46], and other variables (young-adult BMI, weight gain since the age of 20) [46]. Lastly, two articles showed an inverse relationship between BC and HC [43], and body shape at younger ages (6–11 years) [46].

We categorized the studies based on the measure/classification of obesity used, and analyzed their results separately, specifying the classification of obesity adopted by each article (Table S4: Results of the included studies according to the obesity measure/classification used).

### 3.4. Obesity and the Risk of Breast Cancer in Postmenopausal Women

Eleven studies identified an increased risk of BC linked to higher weight [47], BMI ≥ 30 kg/m^2^ [47,49,52], height [40,42], WHR [39,41,43], BMI at 20 years [44], WC [43,46,50], HC, and greatest increase in body shape trajectory [46]. In contrast, five articles observed a non-significant association between BC and BMI [41,50], BMI ≥ 30 kg/m^2^ [40,42,46], height [41,46], and weight [41,42,46]. On the other hand, two articles reported no relationship between BC and BMI at interview [44], and other measures (WHR, young-adult BMI, and weight gain since the age of 20) [46]. Finally, two articles showed an inverse association between BC and HC [43] as well as BMI ≥ 30 kg/m^2^ [45].

**Figure 3:**
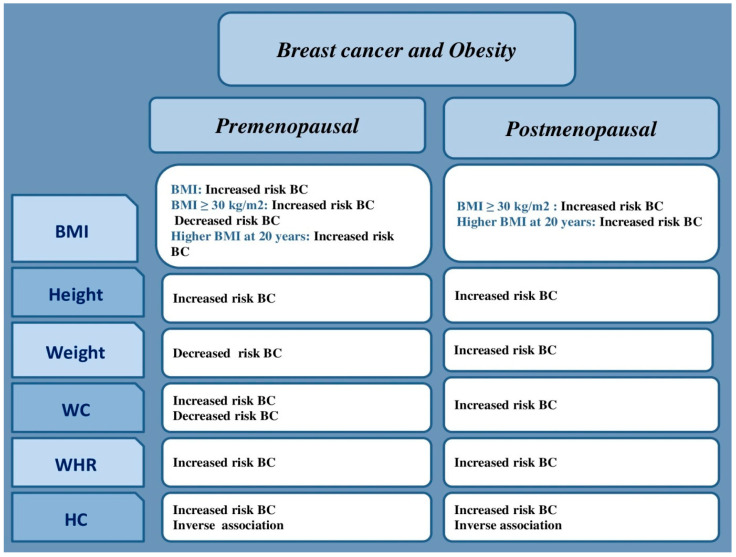
Main significant associations between obesity and risk of pre- and postmenopausal breast cancer risk in Africa.

Figure 3 presents a summary of the main significant associations between obesity and breast cancer risk according to menopausal status.

### 3.5. Quality Assessment and Risk of Bias

In this systematic review, the quality of our selected studies was examined by using the National Institute of Health (NIH): Quality Assessment of Case-Control Studies. The quality assessment checklists of the included case–control studies showed that 10 articles of 15 are good (Table S3: NIH Quality Assessment Tool for case-control Studies in the Supplementary Materials).

## 4. Discussion

To the best of our knowledge, this is the first systematic review to explore the relationship between obesity and the risk of BC among African women.

The link between obesity and BC risk has been widely investigated across various population groups, with notable differences observed based on menopausal status. Studies exploring the link between BMI and premenopausal BC have shown inconsistent results. A meta-analysis conducted in Asian women (including 7593 cases and 14,769 controls) identified a higher risk of BC with increased BMI in premenopausal women [29]; this finding is consistent with the results of our study. In contrast, some studies and meta-analyses have shown that a high BMI decreases the risk of BC in premenopausal women [6,15,23,53,54,55], aligning with our findings. Several meta-analyses and studies have reported an inverse association between BMI and the risk of premenopausal BC [5,6,22,26,27]. However, the findings from the European prospective investigation into cancer and nutrition (EPIC) has indicated a non-significant inverse association between weight, BMI, and premenopausal BC risk [26]. Additionally, the results from a study among Latin American women demonstrated a negative association between weight and premenopausal BC risk [27]. Similar conflicting results have been observed in the current study. The conflicting results may be due to differences in ethnic backgrounds, as ethnicity has been shown to influence insulin resistance and body composition, regardless of family history or hormonal receptor status [32]. Inconsistencies observed in previous studies across ethnic groups may also be linked to variations in body fat distribution among different populations [13]. Additionally, variations in study protocols and designs could play a role in these discrepancies [46].

We found a positive association between increased height and the risk of developing premenopausal BC, which is consistent with a recent cohort study of 125,188 premenopausal Korean women [24]. Similarly, a prospective cohort study (Nurses’ Health Study II), including 108,829 premenopausal women, and a pooled analysis of 20 prospective cohort studies have demonstrated that taller adult height is linked to a higher risk of developing BC [56,57]. In contrast, a pooled analysis of seven prospective cohorts found no significant relationship between height and the risk of premenopausal BC [22]; these findings are in line with our results. These differences may be attributed to the fact that adult height is influenced by a combination of genetic and environmental factors, including environmental conditions, variations in childhood experiences, nutrition, and diseases [58]. Levels of insulin-like growth factor 1 (IGF-1) in both childhood and adulthood are positively associated with adult height in women and are consistently linked to a higher risk of BC [59].

The relationship between central adiposity and the risk of premenopausal BC has given rise to contradictory results in several studies. A recent meta-analysis of 57 studies (26 case–control and 31 prospective cohort), comprising 7,989,315 women, found that central obesity measured by WC was associated with a higher risk of premenopausal BC, with similar results for waist-to-hip ratio [33]. Another meta-analysis indicated that central obesity, as measured by WC but not WHR, was linked to a modestly increased risk of both pre- and postmenopausal BC, independent of BMI [9]. However, a pooled analysis conducted by Harvie et al. suggested that WC and WHR measurements have a minimal influence on the risk of premenopausal BC [60]. A multicenter population-based case–control study found a negative association between adult adiposity and BC risk related to WC [27]. In contrast, a recent meta-analysis of 34 studies reported no association between WC, WHR, and the risk of premenopausal BC, with some studies indicating an inverse relationship between HC and the risk of premenopausal BC [61], which aligns with our findings. Similarly, the Nurses’ Health Study II showed no significant associations between WC, HC, or WHR and the risk of premenopausal BC [32]. These conclusions are consistent with our results.

Our findings showed that the largest change in body shape trajectory was linked to an increased risk of premenopausal BC. Additionally, we found an inverse relationship between body shape at a younger age and the risk of premenopausal BC. This contrasts with a case–control study among Latin American women that found no association between body shape at younger ages or body shape changes over time and the risk of premenopausal BC [27].

The mechanisms underlying these inconsistent associations in premenopausal women are not well understood and remain complex, but they may be partly explained by the distinct roles of total fat and fat distribution in metabolism and their influence on BC development in premenopausal women [32,59]. Another explanation is that WC, in particular, serves only as a single indicator of body fat distribution, and techniques like DEXA (dual-energy X-ray absorptiometry) or bioelectrical impedance, which offer detailed insights into body composition and fat distribution, could be instrumental in clarifying these associations [27].

A strong relationship has been established between higher BMI and an increased risk of BC in postmenopausal women. Previous meta-analyses and studies have found that higher BMI is associated with an increased risk of postmenopausal BC [5,7,15,16,17,18,19,22,26,53,54], which aligns with our findings. Similarly, two more recent meta-analyses showed an increased risk of postmenopausal BC with a higher body mass index [20,61]. Our findings reveal that both weight and height are significantly associated with an increased risk of postmenopausal BC. A pooled analysis also found a positive association between height and the risk of postmenopausal BC [22].

In premenopausal women, estrogen is primarily produced by the ovaries. However, after menopause, most circulating estrogen results from the conversion of adrenal androgens by the aromatase enzyme in adipose tissue [62,63]. Consequently, women with greater body fat levels generally have higher circulating estrogen levels. Furthermore, some studies have shown that obesity is more strongly associated with estrogen receptor (ER)-positive BC than with ER-negative ones [64,65]. Interestingly, postmenopausal women who previously used hormone therapy seem to have a lower obesity-related BC risk [66,67]. These findings provide additional support for the hypothesis that estrogen availability plays a role in postmenopausal BC.

A recent meta-analysis conducted by Chen et al. found that elevated WC and WHR are linked to a higher risk of BC in younger women, both before and after menopause [33]. Additionally, another meta-analysis indicated that central obesity, measured by WC rather than WHR, is associated with a modest increase in the risk of postmenopausal obesity [9]. In contrast, a separate study including Hispanic and non-Hispanic white women demonstrated that postmenopausal WC, HC, and WHR were all positively associated with a higher risk of breast cancer after menopause, independent of BMI [21], which is in line with our findings. Another cohort study in Korean women showed that WC was robustly associated with increased risk for postmenopausal BC, while when accounting for BMI, WC was found to have a negative association with postmenopausal BC risk [30]. The Shanghai Women’s Health Study revealed that the positive association between postmenopausal BC and WC remained significant even after adjusting for BMI, while the association with WHR became non-significant following the same adjustment [68]. We also found a positive association between BMI in early adulthood (at age 20) and both premenopausal and postmenopausal BC, though the results regarding this relationship in postmenopausal women were inconsistent [56,69]. This association may vary depending on the molecular subtypes of BC [70,71].

The underlying pathophysiology of the obesity–BC link is complex and still under investigation. The importance of local and systemic effects of obesity is supported by many studies and involves the following potential mechanisms: increased levels of estrogens, excessive aromatization activity of the adipose tissue, over-expression of pro-inflammatory cytokines, insulin resistance, adipocyte-derived adipokines, hypercholesterolemia, and excessive oxidative stress contribute to the development of BC in obese women, especially in postmenopausal women [72,73,74]. In addition, other hormonal factors associated with obesity, including insulin-like growth factor 1(IGF-1), steroid hormones, AMP-activated protein kinase, and leptin, also play a significant role in the initiation and progression of BC [62,75]. These obesity-related factors can influence tumor initiation, metabolic reprogramming, angiogenesis, progression, and/or response to therapy. Notably, a balanced and healthy diet may help to reduce the expression of these factors [76,77].

Recent data indicate that the incidence of BC is increasing in both premenopausal and postmenopausal women [29]. An explanation for this increase could be lifestyle changes driven by rapid economic development. Over the past 20 years, reduced physical activity and changes in dietary pattern have resulted in a higher average body mass index across the population [78]. Changes in reproductive behaviors can influence sex hormone levels. Combined with the previously discussed lifestyle and dietary factors, these changes might contribute to the increased risk of BC [77,78,79].

The present systematic review is the first to examine the association between obesity and BC risk in native African populations and involves a female population with different demographic, socioeconomic, cultural/behavioral, and anthropometric characteristics that may be different from those of other populations living in other areas [51]. The increasing rates of obesity in Africa highlight the importance of these findings in informing public health policies on cancer prevention, emphasizing that obesity is a modifiable risk factor for BC in African women [47]. There is an urgent need to enhance detection strategies, targeted treatments, and most importantly, to promote primary prevention. In terms of primary prevention, reducing excess weight and increasing physical activity can play a crucial role in reducing the incidence of BC [80].

Our study has limitations. First, several included studies were based on anthropometric measurements that are recorded at the time of BC diagnosis; furthermore, definitions of overweight and obesity differed across the countries represented in the studies, with some studies using BMI categories that did not align with the standardized guidelines set by the WHO. Moreover, a number of the studies relied on BMI measurements obtained through self-reports or questionnaires, whereas the adjustment for key confounding factors associated with BC and obesity varied across the studies in the review. In addition, it was pointed out in the limitations section of some included articles that African women with BC were diagnosed at advanced stages of the disease, which can lead to weight loss. Finally, methodological variations in study protocol and design should be recognized as possible contributors to these conflicting results. Therefore, caution is advised when applying these findings to different contexts. In this study, we did not include gray literature, such as dissertations, conference abstracts, and preprints. We focused on peer-reviewed studies to ensure methodological rigor, but we acknowledge that this approach may have left out valuable research that is not published in academic journals. Expanding the search to sources like OpenGrey and ProQuest Dissertations & Theses in future studies may help to obtain a large picture of the evidence.

Recommendation: A prospective model could offer important insights into the timing of weight gain and its influence on breast cancer risk and prognosis, which would allow a better understanding of the effects and relationship between obesity and breast cancer. To improve comparability between studies, studies should conform to standardized international definitions, such as those of the WHO; additionally, objective anthropometric measurements taken by qualified professionals, rather than relying on self-reported data, should be preferred to avoid potential bias. Standardized adjustment models that include well-established confounders associated with both breast cancer and obesity should be used, analyses should be stratified according to breast cancer stage at diagnosis, and pre-diagnosis weight or BMI trajectories should be used rather than weight at diagnosis alone. Finally, standardized protocols for study design and data collection must be used.

## 5. Conclusions

In this systematic review, BMI was associated with an increased risk of BC in both premenopausal and postmenopausal women. Inconsistent associations have also been found between BMI and BC before and after menopause. WHR was positively associated with BC in pre- and postmenopausal women, while WC showed a positive association with the risk of postmenopausal BC, and inconsistent results with premenopausal BC. Obesity is a modifiable risk factor for breast cancer prevention in African women. However, further prospective studies could provide a more comprehensive insight into this relationship. Therefore, it is recommended that African women maintain a healthy weight and prevent obesity through regular physical activity and a balanced diet.

## Data Availability

Data sharing did not apply to this article because no datasets were generated or analyzed in the current study.

## Supplementary Materials

The following supporting information can be downloaded at: https://www.mdpi.com/article/10.3390/curroncol32030167/s1, Table S1: PRISMA 2020 Checklist. Table S2: Search strategy until 17 February 2025. Table S3: NIH Quality Assessment Tool for case-control Studies. Table S4: Results of the included studies according to the obesity measure/classification used. References [36,38,39,40,41,42,43,44,45,46,47,48,49,50,51,52] were cited in the supplementary materials.

## Abbreviations

The following abbreviations are used in this manuscript:

AICR	American Institute for Cancer Research
AMP-activated protein kinase	Adenosine monophosphate active protein kinase
aOR	Adjusted odds ratio
BC	Breast cancer
BMI	Body mass index
ER	Estrogen receptor
HC	Hip circumference
IGF-1	Insulin-like growth factor1
CIs	Confidence intervals
MeSH	Medical Subject Headings
NIH	National Institute of Health
OR	Odds ratio
PRISMA	Preferred Reporting Items for Systematic Review and Meta-Analysis
RR	Relative risk
WC	Waist circumference
WHR	Waist-to-hip ratio
WHO	World Health Organization
WCRF	World Cancer Research Fund
