# Supplementary material for: Obesity and Risk of Pre- and Postmenopausal Breast Cancer in Africa: A Systematic Review
Source: Curr Oncol. 2025 Mar 14;32(3):167. doi: 10.3390/curroncol32030167 (PMC11941656; doi:10.3390/curroncol32030167)
Supplement: Supplementary file 1 [file curroncol-32-00167-s001.zip › Table S2 Search strategy.pdf]

Table S2: Search strategy until 17 February, 2025

Search number	PubMed (all fields, Filters: Humans)	Results
#4	#1 AND #2 AND #3	1,711
#3	Morocc* OR Tunis* OR Alger* OR Liby* OR Egypt* OR North africa* OR africa northern OR Morocco OR tunisia OR Egypt OR libya OR Algeria OR Algeri* OR Maghr* OR Maroc* OR Arab republic of Egypt OR Maser* OR Égypt* OR South Sudan OR Sudan OR Burundi OR Comoros OR Djibouti OR Eritrea OR Ethiopia OR Kenya OR Madagascar OR Malawi OR Mauritius OR Mozambique OR Rwanda OR Seychelles OR Somalia OR Tanzania OR Uganda OR Zambia OR Zimbabwe OR Benin OR Burkina Faso OR Cabo Verde OR Côte d'Ivoire OR Gambia OR Ghana OR Guinea OR Guinea-Bissau OR Liberia OR Mali OR Mauritania OR Niger OR Nigeria OR Senegal OR Sierra Leone OR Togo OR Angola OR Cameroon OR Central African Republic OR Chad OR Congo OR Democratic Republic of the Congo OR Equatorial Guinea OR Gabon OR Sao Tome and Principe OR Botswana OR Lesotho OR Namibia OR South Africa OR Eswatini OR Africa OR Africa South of the Sahara OR Africa Eastern OR Africa Western OR Africa Southern OR Africa Central OR sub-saharan africa	1,079,608
#2	obesity OR adiposity OR body weight OR obese OR body mass index OR BMI OR body mass OR body size OR overweight OR over-weight OR waist height ratio OR over weight OR body fatness OR body fat OR body composition OR greater birth Weight OR anthropometry measurement OR skin fold measurement OR skin fold thickness OR waist circumference OR hip circumference OR waist to hip ratio OR body fat percentage OR obesity rates OR obesity measurement OR central obesity OR abdominal obesity	1, 599,257
#1	breast cancer OR breast neoplasms OR breast tumor OR lobular breast tumor OR lobular carcinoma OR breast adenocarcinoma OR breast carcinoma OR mammary cancer OR cancer of breast OR breast malignant neoplasms OR carcinoma human mammary OR human mammary neoplasms OR breast malignant tumor OR mammary gland OR female cancer OR contralateral breast Cancer OR premenopausal breast cancer OR postmenopausal breast cancer	2, 253,563
**Search number**	**Web of Science (All fields, without filter)**	**Results**
#4	#1 AND #2 AND #3	990
#3	"africa*" OR "africa" OR "africa eastern" OR "north africa" OR "central africa" OR "south africa" OR "africa western" OR "africa southern" OR "africa south of the sahara" OR "africa northern" OR "north africa" OR "sub-saharan africa"	1, 273,922
#2	"obesity" OR "obes*" OR "adiposity" OR "fat" OR "fatness" OR "body mass index" OR "BMI" OR "body size" OR "Body Weight" OR "weight" OR "overweight" OR "height"	3, 321,745

#1	"breast cancer" OR "breast carcinoma" OR "breast neoplasms" OR "Breast Tumors"	690,351
**Search number**	**SCOPUS (Title/Abstract/Keywords,without filter)**	**Results**
#4	#1 AND #2 AND #3	979
#3	"africa*" OR "africa" OR "africa eastern" OR "north africa" OR "central africa" OR "south africa" OR "africa western" OR "africa southern" OR "africa south of the sahara" OR "africa northern" OR "north africa" OR "sub-saharan africa"	949,431
#2	"obesity" OR "obes*" OR adiposity OR fat OR fatness OR "body mass index" OR BMI OR "body size" OR "Body Weight" OR weight OR overweight OR height	5, 054,702
#1	"breast cancer" OR "breast carcinoma" OR "breast neoplasms" OR "Breast Tumors"	710,312
**Search number**	**Google scholar (title and abstract)**	1,300
	breast cancer|breast neoplasms|premenopausal breast cancer|postmenopausal breast cancer;obesity|adiposity|overweight|body mass index|waist circumference|hip circumference|waist to hip ratio|obesity measurement|central obesity|abdominal obesity;Africa|africa northern|Africa South of the Sahara|Africa Eastern|Africa Western|Africa Southern|Africa Central|sub-saharan africa	1,300
