# Supplementary material for: Obesity and Risk of Pre- and Postmenopausal Breast Cancer in Africa: A Systematic Review
Source: Curr Oncol. 2025 Mar 14;32(3):167. doi: 10.3390/curroncol32030167 (PMC11941656; doi:10.3390/curroncol32030167)
Supplement: Supplementary file 1 [file curroncol-32-00167-s001.zip › Table S3 NIH Quality Assessment.pdf]

Table S3: NIH Quality Assessment Tool for case-control Studies

Author /Reference	Was the research question or objective in this paper clearly stated and appropriate?	Was the study population clearly specified and defined?	Did the authors include a sample size justification?	Were controls selected or recruited from the same or similar population that gave rise to the cases (including the same timeframe)?	Were the definitions, inclusion and exclusion criteria, algorithms or processes used to identify or select cases and controls valid, reliable, and implemented consistently across all study participants	Were the cases clearly defined and differentiated from controls?	If less than 100 percent of eligible cases and/or controls were selected for the study, were the cases and/or controls randomly selected from those eligible?	Was there use of concurrent controls?	Were the investigators able to confirm that the exposure/risk occurred prior to the development of the condition or event that defined a participant as a case?	Were the measures of exposure/risk clearly defined, valid, reliable, and implemented consistently (including the same time period) across all study participants?	Were the assessors of exposure/ risk blinded to the case or control status of participant s?	Were key potential confounding variables measured and adjusted statistically in the analyses? If matching was used, did the investigators account for matching during study analysis?	Overall
**Laamiri FZ et al, 2016, Morocco [38]**	Yes	Yes	No	Yes	Yes	Yes	CD	No	No	No	NA	No	Fair
**Adebamowo CA et al, 2003, Nigeria [39]**	Yes	Yes	No	Yes	Yes	Yes	Yes	No	NR	No	NA	Yes	Good
**Adebamowo CA et al, 2003, Nigeria [40]**	Yes	Yes	No	Yes	Yes	Yes	Yes	No	NR	Yes	NA	Yes	Good
**Okobia MN et al, 2006, Nigeria [41]**	Yes	Yes	No	Yes	Yes	Yes	CD	No	NR	NR	NA	Yes	Fair
**Ogundiran TO et al, 2010, Nigeria [42]**	Yes	Yes	No	Yes	Yes	Yes	Yes	No	No	Yes	NA	Yes	Good
**Ogundiran TO et al, 2012, Nigeria [43]**	Yes	Yes	No	Yes	Yes	Yes	Yes	No	NR	Yes	NA	Yes	Good
**Jordan I et al, 2013,**	Yes	Yes	No	Yes	Yes	Yes	NA	No	Yes	Yes	NA	Yes	Good

**Tanzania [44]**													
**Wang S et al, 2018, Nigeria [45]**	Yes	Yes	No	Yes	Yes	Yes	Yes	No	NR	No	NA	Yes	Fair
**Khalis M et al, 2020, Morroco [46]**	Yes	Yes	No	Yes	Yes	Yes	Yes	No	Yes	Yes	NA	Yes	Good
**Brandão M et al, 2021, Mozambique, Sub-Saharan Africa [47]**	Yes	Yes	No	Yes	Yes	Yes	Yes	No	NR	Yes	NA	Yes	Good
**Akinyemiju T et al, 2021, Nigeria [48]**	Yes	Yes	No	Yes	Yes	Yes	NA	No	NR	Yes	NA	Yes	Good
**Kamal RM et al, 2022, Egypt [49]**	Yes	Yes	No	Yes	Yes	No	NR	No	No	Yes	NA	Yes	Good
**Jacobs I et al, 2022, South Africa [50]**	Yes	Yes	Yes	Yes	Yes	Yes	CD	No	No	Yes	NA	Yes	Good
**Mohammed AM et al, 2023, Egypt [51]**	Yes	Yes	Yes	Yes	Yes	Yes	CD	No	No	Yes	NA	No	Fair
**Oyamienlen CS et al, 2019, Nigeria [52]**	Yes	CD	No	Yes	CD	Yes	CD	No	No	No	NA	No	Fair

NA: Not applicable; NR*: Not reported; CD*: Cannot be determined
