# Supplementary material for: Obesity and Risk of Pre- and Postmenopausal Breast Cancer in Africa: A Systematic Review
Source: Curr Oncol. 2025 Mar 14;32(3):167. doi: 10.3390/curroncol32030167 (PMC11941656; doi:10.3390/curroncol32030167)
Supplement: Supplementary file 1 [file curroncol-32-00167-s001.zip › Table S4 Results according type of anthropometric measurement.pdf]

Table S4: Results of the included studies according to the obesity measure/classification used.

Obesity Measure/Classification	Author, year and country	Results	Classification of Obesity Adopted
BMI	Laamiri FZ et al, 2016, Morocco [38]	**BMI** not been identified as predictive risk of preM BC (OR=0.994;95%CI=0,937-1,05; p= 0,849)	Not mentioned
	Adebamowo CA et al, 2003, Nigeria [40]	**BMI≥30kg/m2:** no association with preM (aOR=1.21; 95%CI=0.56-2.60; p>0.05), and postM BC (aOR=1.82; 95%CI=0.78–4.31; p>0.05)	**BMI** was divided into 4 categories (<18, >18-25, >25-30, and ≥30 kg/m2, Obesity: BMI≥30kg/m2
	Okobia MN et al, 2006, Nigeria [41]	**BMI:** not associated with risk of BC in preM (OR = 0.82, 95% CI 0.49–1.36), and postM BC ((OR = 0.76, 95% CI 0.44–1.32)	Not mentioned
	Ogundiran TO et al, 2010, Nigeria [42]	**BMI≥28 kg/m2:** preM BC (OR=0.70; 95%CI=0.50-0.98; p=0.98), and postM BC (OR=0.76; 95%CI=0.48-1.21; p=0.15)	**BMI** was classified by using cutoff points of <21, 21–23.9, 24–27.9, and ≥28 kg/m2
	JordanI et al, 2013,Tanzania [44]	**Higher BMI at age 20 years:** significant association with preM BC (aOR=1.41; 95%CI=1.10–1.81; p=0.01), and postM BC (aOR=1.38; 95%CI=1.06–1.80; p=0.02)<br>**Higher BMI at interview:** no association with preM BC (aOR=0.94; 95%CI=0.84–1.06; p=0.32), and postM BC (aOR=0.92; 95%CI=0.82-1.03; p=0.14)	Not mentioned
	Wang S et al, 2018, Nigeria [45]	**BMI≥30kg/m2:** preM BC (aOR=0.71; 95%CI=0.57–0.89; p=0.001), postM BC (aOR=0.68; 95%CI=0.52–0.89; p<0.001)	**BMI** was classified (<18,5; 18.5–24.9; 25–29.9; ≥30kg/m)
	Khalis M et al, 2020, Morocco [46]	**BMI>30kg/m2:** not significantly associated with BC risk in either preM (OR=1.78; 95%CI=0.79–4.02; p=0.33),or postM BC(OR=1.64; 95%CI=0.72–3.75; p=0.08)	**BMI** was classified according to the WHO: Underweight women with BMI<18.5 kg/m2, normal weight (< 25 kg/m 2), overweight (25–29 kg/m 2), or obese (≥ 30 kg/m2).
	Brandão M et al, 2021, Mozambique, Sub- Saharan Africa [47]	**Higher BMI:** associated with a higher risk of postM (aOR=1.11; 95%CI=1.04–1.18; p=<0.001),but are protective in preM BC (aOR=0.95; 95%CI=0.91–0.99; p<0.001)	**Obese** if BMI ≥ 30 kg/m2
	Akinyemiju T et al, 2021, Nigeria [48]	In pre/peri-menopausal, but not postM women, **higher BMI >29.8 kg/m 2** was significantly associated with reduced risk of BC	Quartiles of **BMI** were used rather than **WHO** (< 18.5 kg/m 2, underweight, 18.5 - <25 normal weight, 25 - <30 over-weight, and ≥ 30 obese)
	Kamal RM et al, 2022, Egypt [49]	**BMI (≥25):** negative insignificant difference with preM BC (aOR=0.877; 95% CI=0.354–2.170; p=0.776), and statistically significant positive difference with postM BC (aOR=2.280; 95%CI=1.071–4.862; p=0.028)	According to the WHO Classification of **BMI**: obesity (BMI≥ 30 kg/m2), overweight (25 <BMI <30kg/m2) , normal weight (18.5 - <25), underweight (BMI< 18.5 kg/m2)
	Jacobs I et al, 2022, South Africa [50]	**BMI:** no significant association with preM (aOR=1.01; 95%CI=0.56-1.81; p=0.978), or postM BC (aOR=1.18; 95%CI=0.76-1.83; p=0.454)	**BMI:** ≤28·6 kg/m2, 28·6 kg/m2<BMI<34·7 kg/m2, BMI: >34·7 kg/m2, Obesity limited to BMI≥30 kg/m2
	Mohammed AM et al, 2023, Egypt [51]	**BMI:** an increase of BMI by 1%is met by an increase likely of BC by 40.6% in preM BC (aOR=1.406; 95%CI=1.194-1.656; p<0.001)	**BMI:** < 18.5 kg/m 2 = Underweight, 18.5–24.9 kg/m2 = Normal weight, 25–29.9 kg/m. 2 = Overweight, 30–39.9 kg/m2 = Obese, < 40 kg/m.2 = Morbid obese

	Oyamienlen CS et al, 2019, Nigeria [52]	**Higher BMI (more than 30 kg/m 2):** associated with increased risk of preM BC (OR=2.210; 95% CI=1.246-5.970; p=0.120), and postM BC (OR=2.720; 95% CI=1.204-4.054; p=0.010)	**BMI** were categorized according to WHO classification: underweight (BMI< 18.5kg/m 2), normal range (18.5 – 24.9kg/m 2), overweight (25- 30kg/m 2), and obese (BMI≥ 30kg/m 2
**WC**	Okobia MN et al, 2006, Nigeria [41]	**WC:** was not a significant predictor of BC risk in preM women (OR = 1.31, 95% CI 0.83–2.08).	Not mentioned
	Ogundiran TO et al, 2012, Nigeria [43]	**WC(90–138 cm):** preM BC (aOR=2.40; 95%CI=1.52–3.78; p<0.001), and postM BC (OR=2.21; 95%CI=1.25–3.91; p<0.001)	**WC (cm):** 45–73 cm, 74–81 cm 82–89 cm, 90–138 cm.
	Khalis M et al, 2020, Morocco [46]	**Higher WC (≥ 86.0)**:positively associated with preM (aOR=2.92; 95%CI=1.33–6.42; p<0.01), and post M BC (aOR=4.46; 95%CI=1.86–10.66; p<0.01)	**WC (cm)**: T1< 76.6, T2 [76.6–86.0], T3 ≥ 86.0
	Jacobs I et al, 2022, South Africa [50]	**Smaller WC** (≤92 cm): positive association with postMBC(aOR=1·69; 95%CI=1.08-2.63; p=0.020), and nonsignificant association with preM BC (aOR=1.30; 95%CI=0.69-2.44; p=0.406)	**WC (cm):** ≤92 cm, 92 cm < Waist circumference <102 cm, Waist circumference: ≥102 cm
	Mohammed AM et al, 2023, Egypt [51]	**WC:** an increase in WC by 1 cm is associated with a reduction in risk of preM BC by 8.6% (aOR=0.914; 95% CI=0.868–0.963; p=0.001)	**WC (cm):** (< 88 cm, ≥ 88 cm)
**WHR**	Adebamowo CA et al, 2003, Nigeria [39]	**WHR(>0.85):** significant association with postM BC (aOR=2.67; 95%CI=1.05–6.80; p=0.04), no association with preM BC (aOR=1.80; 95%CI=0.85–3.81; p=0.13)	**WHR** was divided into tertiles: ≤0.77, >0.77to≤0.85 ,>0.85
	Okobia MN et al, 2006, Nigeria [41]	**Greater WHR:** positively associated with preM BC (aOR=2.56; 95%CI=1.48-4.41; p<0.05), and postM BC (aOR=2.00; 95%CI=1.04–2.53; p<0.05)	Not mentioned
	Ogundiran TO et al, 2012, Nigeria [43]	**WHR (**≥0.87**):** preM BC (aOR=2.12; 95%CI=1.49-2.99; p<0.001), postM BC (aOR=2.26. 95%CI=1.39–3.68; p<0.001)	**WHR:** <0.77 ,0.77 to<0.81 ,0.81 to<0.87 , ≥0.87
	Khalis M et al, 2020, Morocco [46]	**WHR (≥ 0.89)**: is not significantly associated with BC risk in either preM (OR=1.85, 95%CI=0.88–3.89, p=0.11) or postM BC (2.64, 95%CI= 1.18–5.86, p=0.28).	**WHR:** T1 < 0.83, T2 [0.85–0.90], T3 ≥ 0.89
**HC**	Okobia MN et al, 2006, Nigeria [41]	**HC** were not a significant predictor of BC risk in preM women (OR = 0.73, 95% CI 0.45–1.17),	Not mentioned
	Ogundiran TO et al, 2012, Nigeria [43]	**HC (108–157 cm):** inverse association with preM BC (aOR=0.35; 95%CI=0.22–0.56; p<0.001), and postM BC (aOR=0.38; 95%CI=0.22–0.66; p<0.001).	**HC (cm)**:54–92 cm ,93–99 cm ,100–107 cm ,108–157 cm
	Khalis M et al, 2020, Morocco [46]	**HC (≥ 101.0):** positively associated with preM BC (aOR=3.00; 95%CI:1.42–6.33; p=0.01), and postM BC (aOR=4.08; 95%CI:1.76–9.42; p<0.01)	**HC (cm)**: T1 < 92.6, T2 [92.6-101.0], T3 ≥ 101.0

Abbreviations: breast cancer (BC); body mass index (BMI); waist circumference (WC); waist-to-hip ratio (WHR); hip circumference (HC); odds ratio (OR); adjusted odds ratio (aOR); confidence interval (CI); premenopausal breast cancer (preM BC); postmenopausal breast cancer (postM BC); World Health Organization (WHO).
